# Programmable Carbon Nanotube Networks: Controlling Optical Properties Through Orientation and Interaction

**DOI:** 10.1002/advs.202404694

**Published:** 2024-07-31

**Authors:** Kirill V. Voronin, Georgy A. Ermolaev, Maria G. Burdanova, Aleksandr S. Slavich, Adilet N. Toksumakov, Dmitry I. Yakubovsky, Maksim I. Paukov, Ying Xie, Liu Qian, Daria S. Kopylova, Dmitry V. Krasnikov, Davit A. Ghazaryan, Denis G. Baranov, Alexander I. Chernov, Albert G. Nasibulin, Jin Zhang, Aleksey V. Arsenin, Valentyn Volkov

**Affiliations:** ^1^ Donostia International Physics Center (DIPC) Donostia/San‐Sebastián 20018 Spain; ^2^ Emerging Technologies Research Center XPANCEO Internet City Emmay Tower Dubai United Arab Emirates; ^3^ Moscow Center for Advanced Studies Kulakova str. 20 Moscow Russia; ^4^ Institute Prokhorov General Physics Institute of the Russian Academy of Sciences Moscow 119991 Russia; ^5^ Osipyan Institute of Solid State Physics of the Russian Academy of Sciences Chernogolovka 142432 Russia; ^6^ Beijing National Laboratory for Molecular Sciences College of Chemistry and Molecular Engineering Peking University Beijing 100871 P. R. China; ^7^ Skolkovo Institute of Science and Technology Moscow 121205 Russia; ^8^ Laboratory of Advanced Functional Materials Yerevan State University Yerevan 0025 Armenia; ^9^ Russian Quantum Center Moscow 121205 Russia

**Keywords:** 1D materials, carbon nanotubes, optical anisotropy

## Abstract

The lattice geometry of natural materials and the structural geometry of artificial materials are crucial factors determining their physical properties. Most materials have predetermined geometries that lead to fixed physical characteristics. Here, the demonstration of a carbon nanotube network serves as an example of a system with controllable orientation achieving on‐demand optical properties. Such a network allows programming their optical response depending on the orientation of the constituent carbon nanotubes and leads to the switching of its dielectric tensor from isotropic to anisotropic. Furthermore, it also allows for the achievement of wavelength‐dispersion for their principal optical axes – a recently discovered phenomenon in van der Waals triclinic crystals. The results originate from two unique carbon nanotubes features: uniaxial anisotropy from the well‐defined cylindrical geometry and the intersection interaction among individual carbon nanotubes. The findings demonstrate that shaping the relative orientations of carbon nanotubes or other quasi‐one‐dimensional materials of cylindrical symmetry within a network paves the way to a universal method for the creation of systems with desired optical properties.

## Graphical abstract

This work introduces an innovative approach to programmable materials utilizing carbon nanotubes by controlling their orientation and interaction. This methodology offers enhanced flexibility in tailoring physical properties on demand compared to the twisting strategy employed in 2D materials, owing to the reduction in dimensionality to the 1D case. It enables replicating the recently discovered phenomenon of wavelength‐dispersive optical axes.

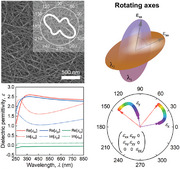

## 1 Introduction

The emergence of artificial material systems^[^ ^1^, ^2^, ^3^ ^]^ with designed properties, such as metamaterials^[^ ^4^ ^]^ and metasurfaces,^[^ ^5^ ^]^ enables a plethora of physical phenomena, such as negative refraction,^[^ ^6^ ^]^ anomalous reflection and refraction,^[^ ^7^ ^]^ and bound states in the continuum,^[^ ^8^ ^]^ to name a few. As a result, countless applications^[^ ^9^, ^10^, ^11^ ^]^ advance rapidly, particularly in the field of nanophotonics, where metamaterials and metasurfaces serve as building blocks for the cutting‐edge technologies like virtual reality,^[^ ^9^ ^]^ invisibility cloaks,^[^ ^10^ ^]^ and machine learning.^[^ ^11^ ^]^

Recent developments in materials science have led to the discovery of extraordinary properties similar to those of the artificial systems, but in natural materials, such as hyperbolic dispersion,^[^ ^12^ ^]^ negative refraction,^[^ ^13^ ^]^ and a giant optical anisotropy.^[^ ^14^ ^]^ In fact, some of the natural materials demonstrate even better optical performance^[^ ^12^, ^15^, ^16^ ^]^ than metamaterials (or metasurfaces) thanks to the atomic origin of their optical properties instead of nanostructuring used to create the desired optical response of artificial systems.^[^ ^1^ ^]^ Furthermore, natural materials themselves can serve as fundamental blocks for fabricating state‐of‐the‐art artificial systems. For example, van der Waals heterostructures provide on‐demand electrical properties^[^ ^17^ ^]^ and optical response^[^ ^18^, ^19^, ^20^ ^]^ if engineered by stacking and twisting of 2D layers with respect to each other at specific angles.

In this regard, carbon nanotubes or other quasi‐one‐dimensional structures bring together the benefits of both natural and artificial materials systems. Indeed, their cylindrical geometry naturally leads to a giant anisotropic response.^[^ ^21^, ^22^ ^]^ At the same time, their properties can be artificially designed by controlling nanotubes angle distribution inside each particular layer within single‐walled carbon nanotubes (SWCNTs) networks.^[^ ^23^ ^]^ Thus, it opens up a unique opportunity to engineer the dielectric tensor of SWCNTs by changing the mutual orientation of SWCNTs from random to partially aligned and to ultimately aligned configurations.^[^ ^21^, ^23^, ^24^, ^25^ ^]^ Though random and aligned carbon nanotube networks are investigated in detail,^[^ ^26^, ^27^ ^]^ the properties of the intermediate state remain undiscovered, and unveiling its optical responses is of great interest for developing on‐demand optical properties in artificial systems. Numerous studies are focused on understanding the intricacies of light–matter interaction, particularly involving SWCNTs and their networks. Techniques such as density functional theory, first‐principle methods, and finite element classical electromagnetic simulations have been employed to calculate the dielectric responses of individual and bundled SWCNTs to electrostatic fields.^[^ ^28^, ^29^, ^30^ ^]^ However, despite these efforts, a systematic investigation into the impact of SWCNTs' mutual orientation on their optical properties remains elusive. To the best of our knowledge, no comprehensive studies have been conducted in this particular area.

In this work, we demonstrate that partial alignment of SWCNT networks may lead to the artificial reconstruction of recently discovered optical effects, such as wavelength‐dispersive principal optical axes in natural materials.^[^ ^31^, ^32^ ^]^ We also provide a theoretical model describing this effect by taking into account individual nanotubes uniaxial polarizability and the interaction among them. These findings confirm the great potential of SWCNT networks for the designing of desired optical responses. In addition, it establishes the importance of the interaction among SWCNTs and offers a straightforward method for the principal optical axes tuning. Given the wide variety of SWCNT networks, our approach may also pave a path toward engineering an on‐demand degree of the rotation of principal optical axes. Therefore, wavelength‐dispersive optical axes in partially aligned SWCNTs present exciting prospects for their utilization in the future adjustable nanophotonic applications.

## 2 Results

### 2.1 Origins of Wavelength‐Dispersive Optical Axes in Single‐Walled Carbon Nanotube Networks

**Figure 1:**
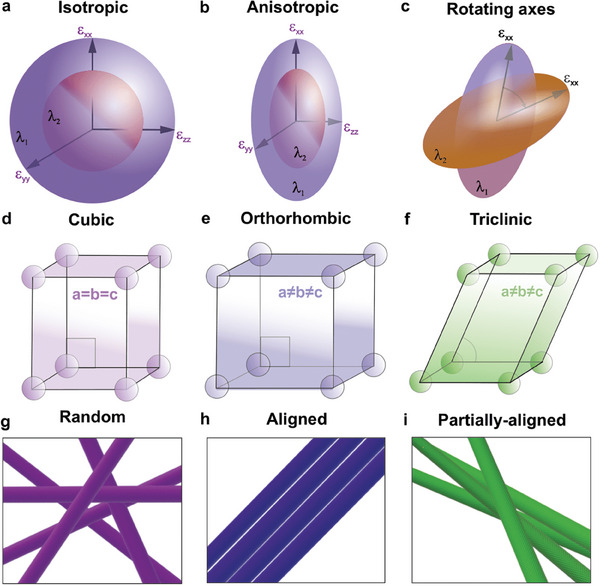
Surfaces of the dielectric functions (optical indicatrix) at two different selected wavelengths for a) isotropic, b) anisotropic, and c) rotating optical axes materials. In natural crystals, they are usually observed in d) cubic, e) orthorhombic, and f) triclinic singonies, respectively. Meanwhile, in artificially designed SWCNT networks, such optical response can be reproduced in g) random, h) aligned, and i) partially aligned configurations.

The distinction between the fixed and wavelength‐dispersive positions of principal optical axes is depicted in **Figure** **1**, which illustrates the concept of the dielectric tensor at various optical wavelengths for materials with isotropic (Figure 1a), anisotropic (Figure 1b), and rotating axes (Figure 1c). In the case of isotropic materials, where ε_*xx*_ = ε_*yy*_, the optical indicatrix possesses rotational symmetry around the optic axis at different wavelengths. The dielectric function, being rotationally symmetric, gives rise to a spherical surface for optical indicatrix, as shown in Figure 1a. This characteristic is common for materials with cubic crystal structures (Figure 1d).

On the other hand, anisotropic materials exhibit different optical responses in different directions. However, the dielectric permittivity tensor of most of the anisotropic materials can be diagonalized in the Cartesian coordinates corresponding to the principal axes of the tensor, and these axes remain stationary across all wavelengths.^[^ ^31^ ^]^ In this case, the optical indicatrix transforms into codirectional ellipsoids, as depicted in Figure 1b. Natural crystals with orthorhombic symmetry serve as an example of optically anisotropic materials with static principal optical axes (Figure 1e). Meanwhile, the nontrivial scenario of the wavelength‐dispersive direction of the principal optical axes (Figure 1c) is possible in triclinic crystals^[^ ^31^, ^32^ ^]^ (Figure 1f). In such materials, the principal axes of the dielectric tensors may differ and exhibit variations with wavelengths, as schematically shown in Figure 1c, which provides an additional optical degree of freedom for the manipulation of light.^[^ ^31^, ^32^ ^]^

Despite its uniqueness, the discovered effect lacks tunability due to the limitations of triclinic crystalline structures. This limitation has sparked interest in engineering artificial materials that exhibit principal optical axis rotation. To achieve this, the desired material must satisfy several criteria. Firstly, it should exhibit a strong optical response, preferably due to the excitonic effects. Secondly, it should possess intrinsically anisotropic properties. Lastly, it should be able to form an extended network of interacting materials. In light of these criteria, individual carbon nanotubes emerge as ideal candidates for building blocks of tunable principal optical axes. They are inherently anisotropic and can form an extended network through interactions with other nanotubes. Indeed, the artificial analogs of natural isotropic, anisotropic, and rotating axis materials can be realized using random (Figure 1g), aligned (Figure 1h), and partially aligned (Figure 1i) nanotubes. In the first two cases, the optical axis remains stationary, while in the case of partially aligned nanotubes, the optical axis exhibits wavelength‐dispersive behavior owing to tube‐to‐tube multi‐exciton interactions (see Supporting Information for details). Noticeably, as we comment below, the electrostatic interaction between the building blocks is crucial for achieving the wavelength‐dispersive optical axes.

**Figure 2:**
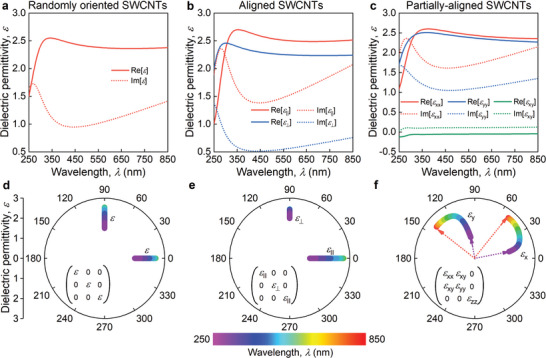
The dielectric tensor of SWCNTs for different configurations of nanotubes. Calculated optical responses for a) random (isotropic dielectric function), b) aligned (anisotropic diagonalized dielectric function), and c) partially aligned SWCNT networks (anisotropic dielectric function with nonzero off‐diagonal elements), and d–f) the corresponding polar view of principal optical axes variation for the Hermitian part of the dielectric tensor. In these polar plots (panels d–f), the angle corresponds to the directions of the principal optical axes basis, for which the dielectric tensor is diagonalized, while the radius shows the dielectric permittivity along the principal optical axes. The arrows in panel (f) represent the main axes of the dielectric permittivity tensor for the wavelength of 250 nm (violet arrows) and 850 nm (red arrows). The insets in (d–f) present corresponding dielectric tensors.

To develop the permittivity tensor model of a carbon nanotube network, we start with an individualized SWCNT as a fundamental building block for the thin film network. To systematically investigate the position of the optical axis, we initially introduced the polarizability tensor of an individual nanotube. Due to the cylindrical symmetry of the nanotube, its polarizability tensor can be divided into components perpendicular to the nanotube's axis (α_⊥_) and parallel to the axis (α_∥_), where α_⊥_ is significantly smaller than α_∥_. By manipulating the orientation of the randomly distributed SWCNTs, we can create aligned or partially aligned networks (**Figure** **2**). This manipulation allows us to achieve different types of dielectric tensors: isotropic, anisotropic with the fixed principal axes, or anisotropic with the wavelength‐dispersive principal axes (Figure 2a–c).

The calculation of the dielectric permittivity tensor of the network composed of nanotubes with a given angular distribution: random (Figure 2a), aligned (Figure 2b), and partially aligned (Figure 2c), is based on the calculation of the electric polarization density inside the film under the external uniform electric field. Namely, we sum up the contributions of the polarizations from each nanotube, whereas the polarization of the single nanotube is calculated within the mean‐field approach. In other words, we consider each nanotube to be characterized by the polarizability^[^ ^21^ ^]^ $\overset{↔}{\alpha }$ and placed at the electric field given by the superposition of the applied external field and the field created by the neighboring nanotubes. For simplicity, by neighboring, we imply only nanotubes that are in contact with the one under consideration (nearest neighbors). Moreover, we consider the contribution to the field from neighboring nanotubes to be localized near the contact of the nanotubes, whereas the freely hanging nanotube sections are assumed to be placed at the uniform external field (see Supporting Information for more details). Importantly, taking into account these fields of neighboring nanotubes is essential for the appearance of the wavelength‐dispersive principle axis phenomenon. Indeed, as shown in Supporting Information, if the polarization of each nanotube depends only on the external field and the properties of this nanotube, but is not affected by the polarizations of the other nanotubes, then the optical response of any network built from these nanotubes can be characterized by the dielectric permittivity tensor with the main axes dependent only on the angular distribution of the nanotubes, but not on the optical properties of the single nanotube. On the other hand, if the polarization of the nanotube is affected also by the polarizations of other nanotubes, the direction of the main axes of the dielectric permittivity tensor of the network become dependent on the polarization tensor of the nanotubes and therefore, the frequency. In other words, the electrostatic interaction between nanotubes makes the main axes of the dielectric permittivity of the nanotube network frequency‐dependent. In our model, we assume the nanotubes to be identical with the same parameters as aligned nanotubes. Although in reality, nanotubes in the network can have different parameters such as length, radius, and chirality, which can affect the properties of the network, especially being correlated with an angular distribution, the assumption in our model that the nanotubes are identical having some average parameters gives satisfactory results. For more precise calculation the model can be improved to consider the distribution of the nanotube parameters, however, our approximation already accurately reproduces experimental results.

### 2.2 Realization of Wavelength‐Dispersive Optical Axes in Single‐Walled Carbon Nanotube Networks

**Figure 3:**
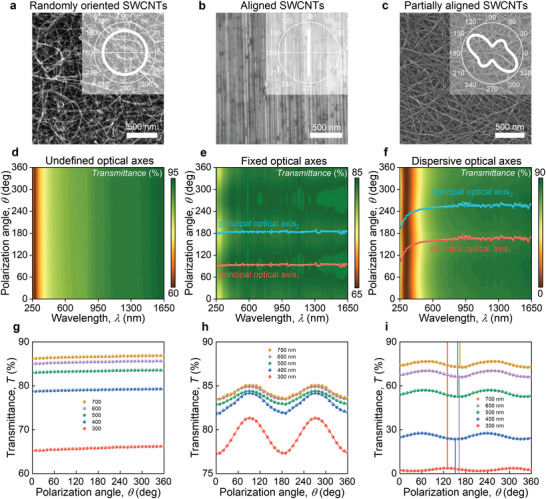
Experimental evidence of optical axes rotation. Scanning electron microscopy images (SEM) show the large area of a) randomly oriented, b) aligned, and c) partially aligned SWCNT networks. The inserts show the distribution of SWCNT directions for each case in polar coordinates. The colormaps of polarized transmittance d–(f) versus wavelength and polarization angle for the three described cases, respectively. The red and cyan points in panels e–f) show the positions of the principal optical axes. These points are obtained through the fitting of polarization‐resolved microtransmittance at each wavelength. g– i) Transmittance versus polarization angle for the selected wavelengths. Lines in panel (i) show the positions of principal optical axes for the selected wavelengths.

In order to verify our concept of programmable carbon nanotube networks for optimized optical axes rotation, we prepared SWCNT films with random (**Figure** **3a**), aligned (Figure 3b), and partially aligned (Figure 3c) orientations of nanotubes. Although the synthesis of random and aligned SWCNTs is well established,^[^ ^33^, ^34^ ^]^ the fabrication of a partially aligned configuration is still a nontrivial task. The natural solution for this challenge is using the substrate morphology, which determines nanotubes' preferential orientation.^[^ ^21^, ^35^ ^]^ In our case, we use the aerosol (floating catalyst) chemical vapor deposition method, where carbon nanotubes are collected on cellulose membrane filters, where the filter pores' heterogeneity can influence the nanotubes' orientation. To verify this hypothesis, we measured the degree of polarization of the cellulose filter in the near‐infrared (NIR) range at various wavelengths, as well as the degree of polarization of SWCNT films with 60% transparency at 550 nm in the optical range, which was transferred from the filter onto a transparent substrate. Figure SI2b (Supporting Information) demonstrates that the filter pores exhibit heterogeneous sizes in different directions. Moreover, we observed that the filter's inhomogeneity impacts the SWCNTs' orientation. Finally, we observed a correspondence between the polarization direction of the nanotubes and that of the filter pores. The corresponding degree of polarization for the filter was measured to be 3%, while for the nanotubes it was 2.3%. This effect may arise due to the influence of the pore direction on the uniformity of the gas flow through the filter, which in turn affects the orientation of the nanotubes (see Supporting Information for more details). Our findings suggest that filters with a high degree of fiber orientation could be utilized to achieve films of oriented SWCNTs.

To quantitatively explore the dependence of alignment on the rotation of the optical axis, we conducted polarization‐dependent transmission measurements along with scanning electron microscopy (SEM) imaging. In Figure 3, we present the results of our measurements. In Figure 3a, we illustrate the percolated network of randomly oriented SWCNTs grown by the aerosol‐based method. To confirm the absence of an optical axis rotation, we performed polarized transmission measurements. The results are shown in Figure 3d, where the transmittance is plotted as a function of wavelength (λ) and polarization angle (θ). Notably, at lower wavelengths, we observe a monotonic decrease in transmission due to π‐plasmon absorption. Importantly, this response is independent of the polarization angle, indicating the film's isotropic properties (Figure 3g). Therefore, we conclude that no optical axis rotation is present in this sample (Figure 3d). Next, in Figure 3b, we present a SEM image of a large area of densely packed SWCNTs, displaying an ordered structure with perfect alignment. To investigate the anisotropy dependence and optical axis rotation in this aligned sample, like the previous case, we plot the transmittance in Figure 3e as a function of wavelength (λ) and polarization angle (θ). As expected, we observe a significant optical anisotropy (Figure 3h) evidenced by the sinusoidal behavior upon changing the polarization angle.

Finally, we delve into the transmission behavior of our partially aligned SWCNTs, as demonstrated in Figure 3c. Similar to the previous sample, transmittance (Figure 3f) decreases toward lower wavelengths and exhibits sinusoidal dependence on polarization angle (Figure 3i). Furthermore, we observe a monotonic principal optical axes rotation effect in accordance with our theoretical predictions (see Supporting Information). Thus, our experiments presented in Figure 3 serve as a proof‐of‐the concept confirming that the angular distribution of single‐walled carbon nanotubes allows on‐demand programming of their optical properties.

## 3 Discussion

Recent studies^[^ ^31^, ^32^ ^]^ demonstrated the emergence of an intriguing and novel optical effect of wavelength‐dispersive directions of the principal optical axes in crystalline materials. As a matter of fact, controlling and engineering the rotational degree of the optical axes in crystals still remains a significant challenge. Here, we present one of the possible approaches aimed to resolve this issue, which is based on artificial reconstruction of orientations of excitons in anisotropic one‐dimensional materials with well‐defined compositions and orientations. Our particular study is focused on SWCNTs in particular as they offer a distinct advantage and can form extended networks arranged in predictable and controlled manners.^[^ ^36^ ^]^ Given these benefits, we conducted a comprehensive and quantitative analysis of optical axis rotation, unveiling the underlying physical mechanisms using partially aligned SWCNTs with various mutual orientations.

The optimization of the optical axis rotation effect in SWCNTs holds significant promise for various applications. For example, controlled optical axes rotation can result in wavelength‐switchable propagation directions of waveguide modes.^[^ ^31^ ^]^ Moreover, dispersive optical axes can enhance the sensitivity of topological biosensors owing to the rapid change of substrate optical response due to switching from one optical axis to another optical axis with slight modification of analyte's refractive index.^[^ ^37^ ^]^ Finally, one can implement our SWCNTs films with rotating optical axes for anticounterfeiting labeling using the positions of optical axes for identification since to date there are no other options to control optical axes.^[^ ^38^ ^]^ By programming the geometry and metallicity of SWCNTs, varying their diameters,^[^ ^39^ ^]^ conductivity type,^[^ ^40^, ^41^ ^]^ and lengths,^[^ ^42^ ^]^ one can precisely tailor the wavelength at which the rotation effect exhibits its maximal impact. Precise tuning of optical properties, mainly band gap of SWCNTs, can be obtained by filling their hollow diameter.^[^ ^43^ ^]^ Moreover, similar approach can be used to significantly broaden the functionality of the material.^[^ ^44^, ^45^ ^]^ Besides, we leveraged only the fundamental properties of SWCNTs to achieve wavelength‐dispersive optical axes. Hence, our approach can be potentially applied to the systems beyond SWCNTs, including multiwalled, transition metal dichalcogenide‐based, and even heterostructured nanotubes.^[^ ^46^ ^]^ As a result, the control of the orientation of individual nanotubes provides means to tailor their optical properties for specific applications opening up opportunities for advancements in various fields, such as photonics, optoelectronics, and nanotechnology in general.

## 4 Experimental Section

### Sample Preparation

Randomly and partially aligned SWCNTs with average 1.8 nm diameters were synthesized using the aerosol (floating catalyst) chemical vapor deposition method^[^ ^47^ ^]^ based on CO decomposition via the Boudouard reaction on the surface of Fe‐based catalyst aerosol particles. Aligned nanotubes with average diameter of 1.3–1.8 nm were grown on ST‐cut quartz substrates using multicycle loading of copper catalysts.^[^ ^34^ ^]^

### Morphology Characterization

The structural morphology of the deposited NT films was visualized via scanning electron microscopy (SEM, JSM70001F, JEOL, Tokyo, Japan) working in the secondary electron imaging mode with a Schottky emitter, an acceleration voltage of 30 kV and a working distance of approximately 7 mm.

### Spectroscopy Measurements

The polarized microtransmittance measurement technique implemented on Accurion nanofilm_ep4 ellipsometer was used to determine the principal optical axes. Microtransmittance measurements were performed using x7 objective in a broad spectral range 250–1700 nm with 5 nm step. During the measurements, the polarizer and analyzer of the ellipsometer was aligned and the obtained polarized microtransmittance for each wavelength was fitted by the expression:

(1) $$\begin{array}{ccc} T\;\left(\theta ,\lambda \right) & = & a^{2}\;\cos^{4}\left(\theta -\varphi \right)+b^{2}\sin^{4}\left(\theta -\varphi \right)\\ & & +2ab\cos^{2}\left(\theta -\varphi \right)\sin^{2}\left(\theta -\varphi \right)\cos\left(\Delta \psi \right) \end{array}$$

where *T*(θ, λ) is the polarized microtransmittance, which depends on the polarizer's/analyzer's angle θ, and the incident wavelength λ. The *a* ^2^ and *b* ^2^ are the transmittances of beams polarized along in‐plane principal optical axes, Δψ is the phase difference between transmitted rays polarized along principal optical axes, and φ is the the angular position of the principal optical axis (see cyan points in Figure 3e,f), whereas another principal optical axis is given by sum φ + 90°. Coupled analyzer and polarizer in parallel configuration were rotated in the whole circle from 0° to 360° in 10° step. The transmitted light was collected from a spot of 200 µm diameter.

## Conflict of Interest

The authors declare no conflict of interest.

## Author Contributions

K.V.V., G.A.E. and M.G.B. contributed equally to this work. G.A.E., M.G.B., A.I.C., A.G.N., J.Z., A.V.A., and V.S.V. suggested and directed the project. G.A.E., M.G.B., A.S.S., D.I.Y., M.I.P., and D.S.K. performed the measurements and analyzed the data. A.N.T., Y.X., L.Q., D.V.K., and D.A.G. prepared the samples. K.V.V. and D.G.B. provided theoretical support. K.V.V., G.A.E., and M.G.B. wrote the original manuscript. All authors reviewed and edited the paper. All authors contributed to the discussions and commented on the paper.

## Supporting information

Supporting Information

## Data Availability

The data that support the findings of this study are available from the corresponding author upon reasonable request.
