# Supplementary material for: Programmable Carbon Nanotube Networks: Controlling Optical Properties Through Orientation and Interaction
Source: Adv Sci (Weinh). 2024 Jul 31;11(36):2404694. doi: 10.1002/advs.202404694 (PMC11422810; doi:10.1002/advs.202404694)
Supplement: Supplementary file 1 — Supporting Information [file ADVS-11-2404694-s001.pdf]

ADVANCED SCIENCE
Open Access

Supporting Information

for *Adv. Sci.*, DOI 10.1002/advs.202404694

Programmable Carbon Nanotube Networks: Controlling Optical Properties Through Orientation and Interaction

*Kirill V. Voronin, Georgy A. Ermolaev, Maria G. Burdanova, Aleksandr S. Slavich, Adilet N. Toksumakov, Dmitry I. Yakubovsky, Maksim I. Paukov, Ying Xie, Liu Qian, Daria S. Kopylova, Dmitry V. Krasnikov, Davit A. Ghazaryan, Denis G. Baranov, Alexander I. Chernov, Albert G. Nasibulin, Jin Zhang, Aleksey V. Arsenin and Valentyn Volkov**

Supporting Information for: Programmable carbon nanotube networks: controlling optical properties through orientation and interaction

Kirill V. Voronin[1†], Georgy A. Ermolaev[2†], Maria G. Burdanova[3,4,5†], Aleksandr S. Slavich[3], Adilet N. Toksumakov[3], Dmitry I. Yakubovsky[3], Maksim I. Paukov[3], Ying Xie[6], Liu Qian[6], Daria S. Kopylova[7], Dmitry V. Krasnikov[7], Davit A. Ghazaryan[3,8], Denis G. Baranov[3], Alexander I. Chernov[3,9], Albert G. Nasibulin[7], Jin Zhang[6], Aleksey V. Arsenin[2,8], Valentyn Volkov[2,8*]

[1]Donostia International Physics Center (DIPC), Donostia/San-Sebastián, 20018, Spain.
[2]Emerging Technologies Research Center, XPANCEO, Dubai, 00000, United Arab Emirates.
[3]Moscow Center for Advanced Studies, Kulakova str. 20, Moscow, 123592, , Russia.
[4]Prokhorov General Physics Institute of the Russian Academy of Sciences, , Moscow, 119991, Russia.
[5]Osipyan Institute of Solid State Physics of the Russian Academy of Sciences, , Chernogolovka, 142432, , Russia.
[6]Beijing National Laboratory for Molecular Sciences, College of Chemistry and Molecular Engineering Peking University, Beijing, 100871, P. R. China.
[7]Skolkovo Institute of Science and Technology, Moscow, 121205, Russia.
[8]Laboratory of Advanced Functional Materials, Yerevan State University, Yerevan, 0025, Armenia.
[9]Russian Quantum Center, Moscow, 121205, Russia.

*Corresponding author(s). E-mail(s): vsv@xpanceo.com;

Contributing authors: kirill.voronin@dipc.org;
ermolaev-georgy@xpanceo.com; burdanova.mg@mipt.ru;
slavich.as@phystech.edu; adilet.toksumakov@phystech.edu;
dmitrii-y@mail.ru; paukov.mg@phystech.edu; 2306891010@pku.edu.cn;
qianliu-cnc@pku.edu.cn; d.kopylova@skoltech.ru; d.Krasnikov@skol.tech;
dav280892@gmail.com; baranov.mipt@gmail.com; a.chernov@rqc.ru;
a.nasibulin@skol.tech; jinzhang@pku.edu.cn; arsenin@xpanceo.com;
†These authors contributed equally to this work.

Calculation of the dielectric permittivity tensor of the film composed of the nanotubes

To calculate the dielectric permittivity tensor of the layer of weakly interacting nanotubes, we assume that its optical response consists of two contributions: the response of the free-standing nanotubes, $\overleftrightarrow{\varepsilon}_0$, and the response of the intersections of nanotubes, $\overleftrightarrow{\varepsilon}_i$, that is,

$$\overleftrightarrow{\varepsilon} = \overleftrightarrow{\varepsilon}_0 + \overleftrightarrow{\varepsilon}_i. \tag{1}$$

First, we estimate the response of the free-standing nanotubes as follows,

$$\overleftrightarrow{\varepsilon}_0 = \int_0^{\pi} g(\varphi) \overleftrightarrow{\varepsilon}_a(\varphi) d\varphi, \tag{2}$$

where $\overleftrightarrow{\varepsilon}_a(\varphi)$ is the dielectric permittivity tensor of aligned nanotubes in coordinates rotated by the angle φ with respect to its main axis, and $g(\varphi)$ is the angular distribution of the nanotubes in the layer. In other words, linearity allows us to sum up the contribution from the nanotubes oriented in each direction with the corresponding weights. The rotation transformation of the dielectric permittivity tensor reads,

$$\begin{aligned}\overleftrightarrow{\varepsilon}_a(\varphi) &= \begin{pmatrix} \cos\varphi & -\sin\varphi \\ \sin\varphi & \cos\varphi \end{pmatrix} \begin{pmatrix} \varepsilon_{\parallel} & 0 \\ 0 & \varepsilon_{\perp} \end{pmatrix} \begin{pmatrix} \cos\varphi & \sin\varphi \\ -\sin\varphi & \cos\varphi \end{pmatrix} = \\ &= \begin{pmatrix} \varepsilon_{\parallel}\cos^2\varphi + \varepsilon_{\perp}\sin^2\varphi & (\varepsilon_{\parallel} - \varepsilon_{\perp})\sin\varphi\cos\varphi \\ (\varepsilon_{\parallel} - \varepsilon_{\perp})\sin\varphi\cos\varphi & \varepsilon_{\perp}\cos^2\varphi + \varepsilon_{\parallel}\sin^2\varphi \end{pmatrix}.\end{aligned} \tag{3}$$

Therefore,

$$\overleftrightarrow{\varepsilon}_0 = \int_0^{\pi} g(\varphi) \begin{pmatrix} \varepsilon_{\parallel}\cos^2\varphi + \varepsilon_{\perp}\sin^2\varphi & (\varepsilon_{\parallel} - \varepsilon_{\perp})\sin\varphi\cos\varphi \\ (\varepsilon_{\parallel} - \varepsilon_{\perp})\sin\varphi\cos\varphi & \varepsilon_{\perp}\cos^2\varphi + \varepsilon_{\parallel}\sin^2\varphi \end{pmatrix} d\varphi. \tag{4}$$

Second, we estimate the interaction term $\overleftrightarrow{\varepsilon}_i$. For this, we consider the polarization of two nanotubes intersecting at an angle ϕ, see Fig. (1)a. Due to the presence of

the mirror symmetry relative to two lines along the bisector of the angle formed by nanotubes, this system has a diagonal polarizability tensor in the axes shown in Fig. 1, which can be written as $\beta = \mathrm{diag}(\beta_x, \beta_y)$. To determine this polarizability, we apply the external electric field $\mathbf{E}_0$ along the x-axis. Both nanotubes acquire similar magnitude polarizations, $p_{\|}$ and $p_{\perp}$ along the nanotube and perpendicular to it respectively, that can be written as follows,

$$\begin{cases} p_{\|} = \alpha_{\|} E_0 \cos\frac{\phi}{2} + \alpha_{\|}\Gamma\left(p_{\|}\cos\phi + p_{\perp}\sin\phi\right) \\ p_{\perp} = \alpha_{\perp} E_0 \sin\frac{\phi}{2} + \alpha_{\perp}\Gamma\left(p_{\|}\sin\phi + p_{\perp}\cos\phi\right), \end{cases} \tag{5}$$

where Γ is a phenomenological constant relating the field created by the one nanotube at the position of the second nanotube, which we consider to be a fitting parameter. Solving system (5), we obtain

$$p_{\|} = \frac{\alpha_{\|}(1+\alpha_{\perp}\Gamma)E_0\cos\frac{\phi}{2}}{1-\alpha_{\|}\alpha_{\perp}\Gamma^2-(\alpha_{\|}-\alpha_{\perp})\Gamma\cos\phi} \tag{6}$$

$$p_{\perp} = \frac{\alpha_{\perp}(1+\alpha_{\|}\Gamma)E_0\sin\frac{\phi}{2}}{1-\alpha_{\|}\alpha_{\perp}\Gamma^2-(\alpha_{\|}-\alpha_{\perp})\Gamma\cos\phi} \tag{7}$$

Summing the contributions from both nanotubes, we obtain the polarization of the whole system,

$$P_x = \frac{2E_0\left(\alpha_{\|}\cos^2\frac{\phi}{2}+\alpha_{\perp}\sin^2\frac{\phi}{2}+\alpha_{\|}\alpha_{\perp}\Gamma\right)}{1-\alpha_{\|}\alpha_{\perp}\Gamma^2-(\alpha_{\|}-\alpha_{\perp})\Gamma\cos\phi}. \tag{8}$$

Therefore, the polarizability of the nanotube intersection can be found as $\beta_x = P_x/E_0$. We estimate the polarization of the nanotube by the dielectric permittivity of the aligned nanotubes, namely, $\alpha_{\|} \propto \varepsilon_{\|} - 1$, and $\alpha_{\perp} \propto \varepsilon_{\perp} - 1$, as a result, the polarization of the nanotubes interaction reads,

$$\beta_x \propto \varepsilon_{xeff}(\phi) := \frac{\varepsilon_{\|}\cos^2\frac{\phi}{2}+\varepsilon_{\perp}\sin^2\frac{\phi}{2}-1+(\varepsilon_{\|}-1)(\varepsilon_{\perp}-1)\gamma}{1-(\varepsilon_{\|}-1)(\varepsilon_{\perp}-1)\gamma^2-(\varepsilon_{\|}-\varepsilon_{\perp})\gamma\cos\phi}, \tag{9}$$

where γ is the dimensionless interaction coefficient. Due to the symmetry, β_y is given by the same equation but with the changed angle, $\phi \to \pi - \phi$, that is,

$$\beta_y \propto \varepsilon_{yeff}(\phi) := \frac{\varepsilon_{\|}\sin^2\frac{\phi}{2}+\varepsilon_{\perp}\cos^2\frac{\phi}{2}-1+(\varepsilon_{\|}-1)(\varepsilon_{\perp}-1)\gamma}{1-(\varepsilon_{\|}-1)(\varepsilon_{\perp}-1)\gamma^2+(\varepsilon_{\|}-\varepsilon_{\perp})\gamma\cos\phi}, \tag{10}$$

Integrating contributions from all intersections we obtain the interaction contribution to the dielectric permittivity of the layer:

$$\overleftrightarrow{\varepsilon}_i = \zeta\int_0^{\frac{\pi}{2}} d\varphi \int_0^{\frac{\pi}{2}} d\phi\, g\left(\varphi-\frac{\phi}{2}\right) g\left(\varphi+\frac{\phi}{2}\right)\sin\phi\times$$

$$\times \begin{pmatrix} \varepsilon_{xeff}(\phi)\cos^2\varphi + \varepsilon_{yeff}(\phi)\sin^2\varphi & (\varepsilon_{xeff}(\phi) - \varepsilon_{yeff}(\phi))\sin\varphi\cos\varphi \\ (\varepsilon_{xeff}(\phi) - \varepsilon_{yeff}(\phi))\sin\varphi\cos\varphi & \varepsilon_{yeff}(\phi)\cos^2\varphi + \varepsilon_{xeff}(\phi)\sin^2\varphi \end{pmatrix}. \quad (11)$$

where ζ is the phenomenological constant related to the intersection density, which characterizes how much the intersections contribute to the full dielectric permittivity tensor. In Eq. (11) the integral over ϕ corresponds to the summation of all possible intersection angles, and the integral over φ corresponds to the summation of all possible intersection orientations. The intersection distribution, $g\left(\varphi - \frac{\phi}{2}\right) g\left(\varphi + \frac{\phi}{2}\right) \sin\phi$ is the multiplication of the densities of the nanotubes oriented in the way providing the considered intersection and the term $\sin\phi$, which is proportional to the number of intersections each nanotube participates in (see Fig. 1b-d).

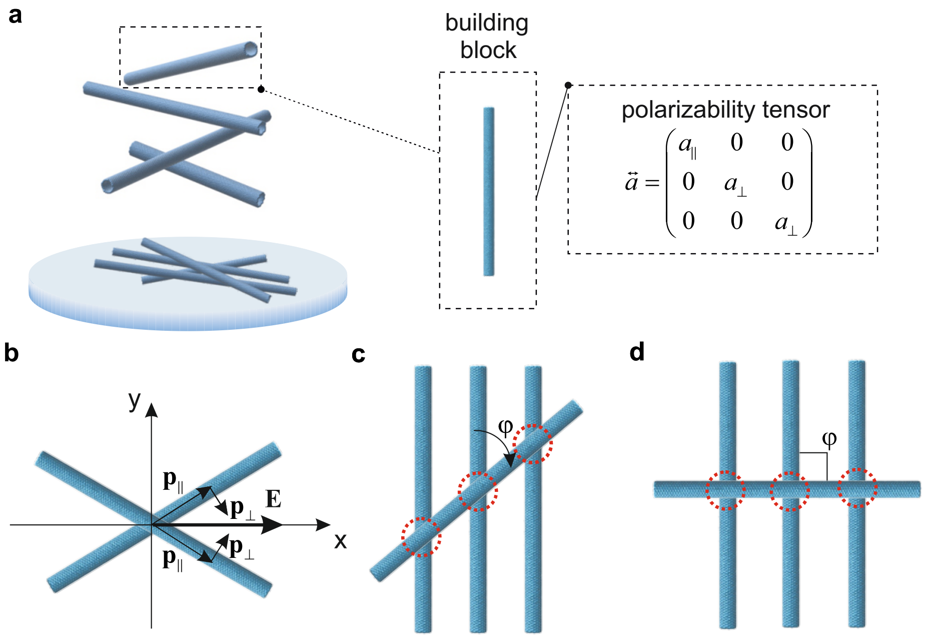

Fig. 1 (a) Individual CNT in the network can be considered as a single building block described by a diagonal polarizability tensor with two identical elements due to the anisotropic properties. (b) The intersection of two nanotubes in the external electric field. (c) Intersections at different angles

Note, if we neglect the interaction between nanotubes, in other words, set ζ to be equal to zero, the principal axes of the dielectric permittivity tensor become independent of frequency. To show this, we write the tensor $\overleftrightarrow{\varepsilon}_0$ in a rotated coordinate system. For this, we notice that the rotation of the coordinate system on the angle θ is equivalent to the change of the angular distribution function, $g(\varphi) \to g(\varphi + \theta)$. If the tensor is diagonal in this coordinate system, out of diagonal components are equal

to zero, then taking into account Eq. (4) we write

$$\int_0^\pi g(\varphi+\theta)(\varepsilon_\parallel-\varepsilon_\perp)\sin\varphi\cos\varphi d\varphi = \frac{\varepsilon_\parallel-\varepsilon_\perp}{2}\int_0^\pi g(\varphi+\theta)\sin 2\varphi d\varphi = 0. \tag{12}$$

As $g(\varphi)$ is π-periodic it can be represented as a Fourier series,

$$g(\varphi) = c_0 + \sum_{n=1}^{\infty} a_n \sin(2n\varphi) + b_n\cos(2n\varphi) = c_0 + \sum_{n=1}^{\infty} c_n\cos(2n\varphi+\theta_n). \tag{13}$$

Equation (12) is satisfied if we choose $\theta = -\frac{\theta_1}{2}$, because of the orthogonality of the functions $\{1, \sin(2\varphi), \cos(2\varphi), \sin(4\varphi), \cos(4\varphi), ...\}$ on the inerval $[0,\pi]$. Thus, the direction of the principal axes is determined by the distribution function and independent of the frequency. On the other hand, in the general case, the tensor including interaction is not diagonal for a fixed basis.

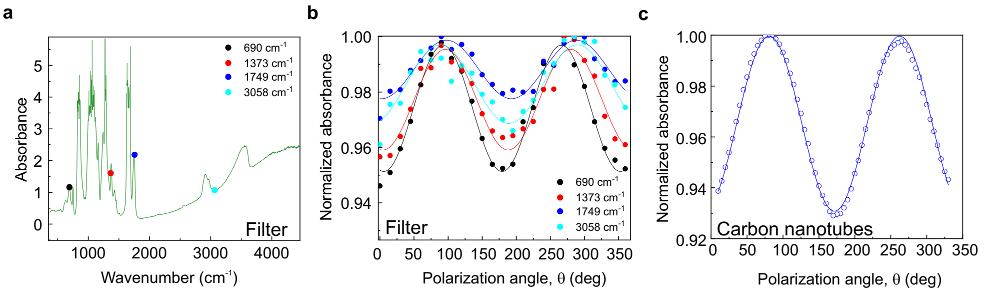

Fig. 2 The evidence of the partial alignment of membranes. (a) The broadband absorbance spectra (-$log_{10}(T)$) of the filter with sharp absorption lines. (b) The absorbance versus polarization angle verifies the partial alignment of the membrane via sinusoidal shape. (b) CNT mimic the shape of the filter and showed similar dependence of the Absorbance versus polarization angle.

The simplest illustrative example is nanotubes aligned along two directions with the angles of 0 and θ relative to the x-axis: $g(\varphi) = a_1\delta(\varphi+\theta/2) + a_2\delta(\varphi-\theta/2)$. Performing integration in Eq. (4) and Eq. (11), we obtain the following expression for the dielectric permittivity tensor of the layer.

$$\overleftrightarrow{\varepsilon} = (a_1+a_2)\begin{pmatrix} \varepsilon_\parallel\cos^2\frac{\theta}{2}+\varepsilon_\perp\sin^2\frac{\theta}{2} & \frac{a_2-a_1}{a_1+a_2}(\varepsilon_\parallel-\varepsilon_\perp)\sin\frac{\theta}{2}\cos\frac{\theta}{2} \\ \frac{a_2-a_1}{a_1+a_2}(\varepsilon_\parallel-\varepsilon_\perp)\sin\frac{\theta}{2}\cos\frac{\theta}{2} & \varepsilon_\perp\cos^2\frac{\theta}{2}+\varepsilon_\parallel\sin^2\frac{\theta}{2}\end{pmatrix} + \\ + 4a_1a_2\zeta\sin\theta\begin{pmatrix}\varepsilon_{xeff}(\theta) & 0 \\ 0 & \varepsilon_{yeff}(\theta)\end{pmatrix}. \tag{14}$$

Note, if $a_1 = a_2$ tensor Eq. (14) becomes diagonal, because of the appearance of the mirror symmetries.

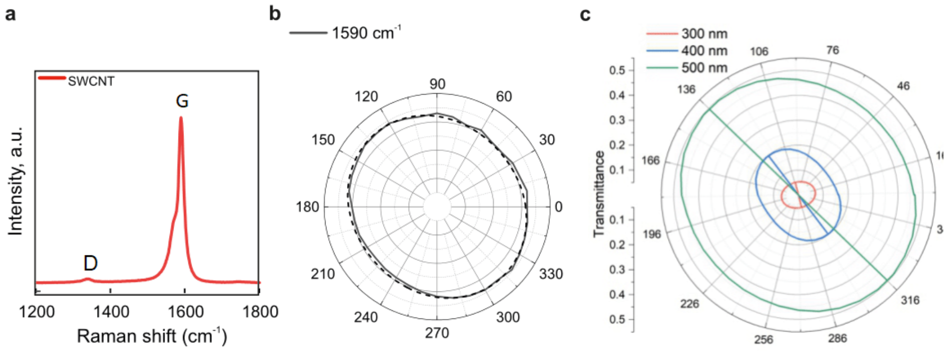

Fig. 3 (a) Raman spectrum of the partially-aligned SWCNTs. Two distinct Raman scattering peaks including the D band and the G band are observed. (b) Polar plots of the Raman intensity 1590 cm^{-1} and (c) transmittance at 300, 400 and 500 nm.

Sample preparation

Randomly and partially-aligned SWCNTs with 1.8 nm were synthesized using the aerosol (floating catalyst) chemical vapor deposition method [1] based on CO decomposition via the Boudouard reaction on the surface of Fe-based catalyst aerosol particles. Aligned nanotubes with the average diameter of 1.2-1.7 nm were prepared using the ST-cut quartz substrates using the multi-cycle loading of copper catalysts[2]. The morphology of nanotubes were characterized by the scanning electron microscopy (SEM).

We measured the degree of polarization of the cellulose filter in the near-infrared (NIR) range at various wavelengths, as well as the degree of polarization of SWCNT (single-walled carbon nanotube) films with 60 % transparency at 550 nm in the optical range, which was transferred from this filter onto a transparent substrate. Fig. 2b demonstrates that the filter pores exhibit heterogeneous sizes in different directions. Moreover, we observed that the filter's inhomogeneity impacts the SWCNTs' orientation.

Polarization dependent Raman and transmission spectra

To quantitatively explore the dependence of alignment on rotation of optical axis, we used TEM together with the polarisation-dependent Raman and transmission spectroscopy measurements (SI Fig. 3). The observed Raman features depend on the representative polarization of the incident and scattered light, which is typical for one-dimensional materials. Two prominent features are D- and G- bands at 1340 and 1590 cm-1. These modes strongly depend on the polarization angle and exhibit a similar polarization dependence. Moreover, the similarity between the maximum intensity and angle for both transmission and Raman spectroscopy results suggests that the main reason for optical axis rotation comes from the alignment of SWCNT.

Transmission and Scanning Electron Microscopy images

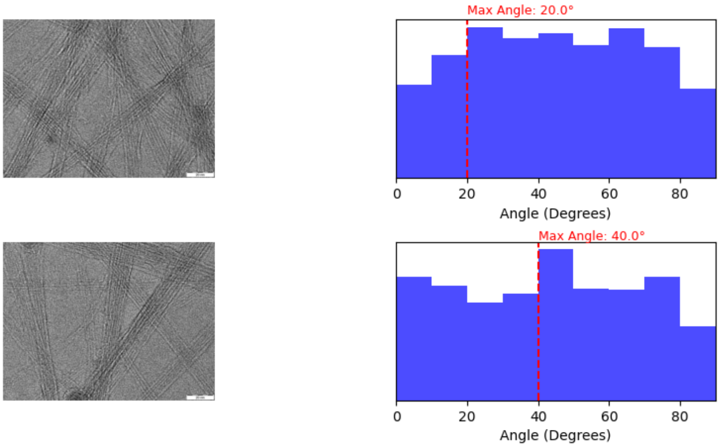

Fig. 4 Additional TEM images reveal the SWCNT network's structure. The angle distribution chart provides a statistical analysis of the SWCNTs' alignment within the network.

Additional TEM images of the SWCNT network show the detailed structure of the network (Fig. 5-**??**). We mark the main direction of the network's fibers, which points the main orientation of the SWCNTs. The angle distribution chart demonstrates a statistical analysis of the SWCNTs' orientation within the network. Fig. 30-33 show the additional SEM images of the nitrocellulose membranes used in the CNTs' synthesis. The SEM method does not allow precisely determine the alignment of the cellulose membrane, so we used spectroscopic methods present in SI.

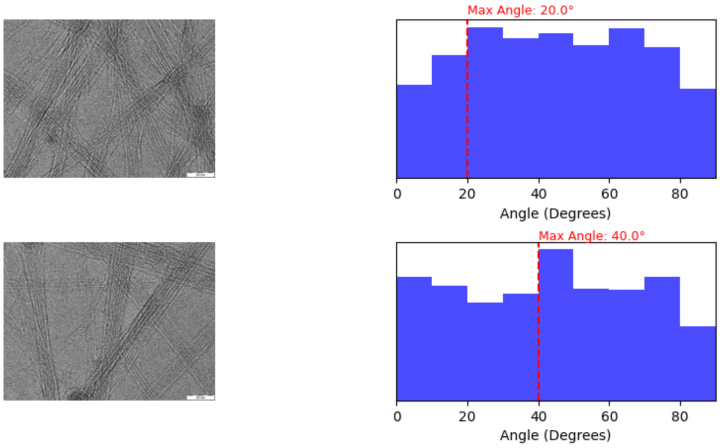

Fig. 5 Continue. Additional TEM images reveal the SWCNT network's structure. The angle distribution chart provides a statistical analysis of the SWCNTs' alignment within the network.

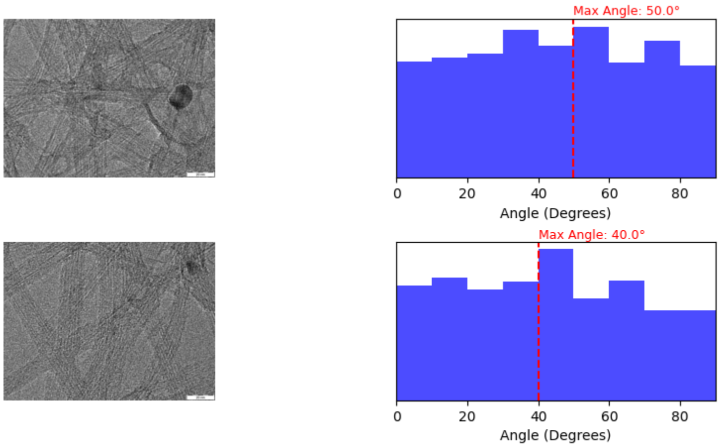

Fig. 6 Continue. Additional TEM images reveal the SWCNT network's structure. The angle distribution chart provides a statistical analysis of the SWCNTs' alignment within the network.

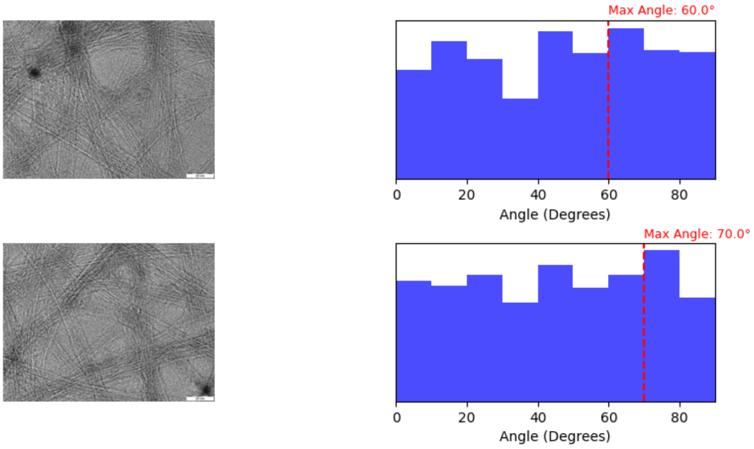

Fig. 7 Continue. Additional TEM images reveal the SWCNT network's structure. The angle distribution chart provides a statistical analysis of the SWCNTs' alignment within the network.

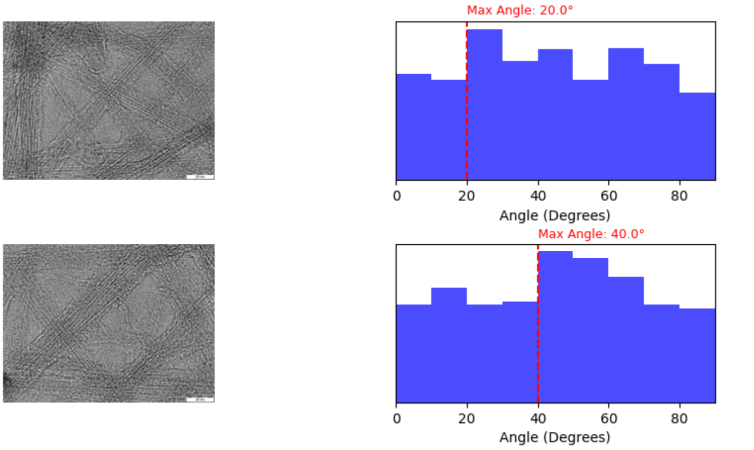

Fig. 8 Continue. Additional TEM images reveal the SWCNT network's structure. Additional TEM images reveal the SWCNT network's structure. The angle distribution chart provides a statistical analysis of the SWCNTs' alignment within the network.

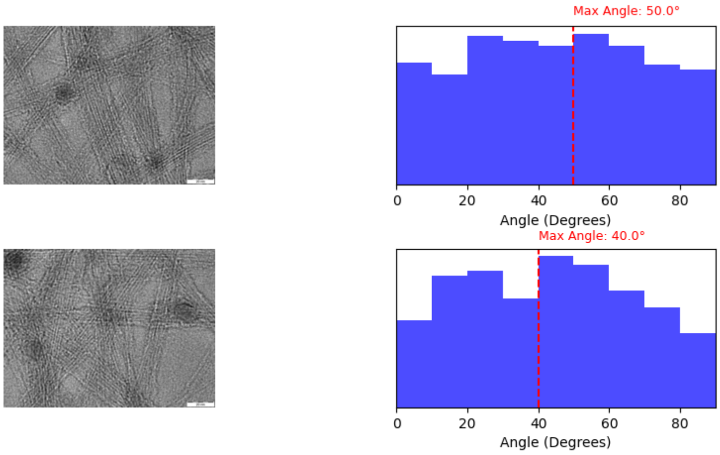

Fig. 9 Continue. Additional TEM images reveal the SWCNT network's structure. The angle distribution chart provides a statistical analysis of the SWCNTs' alignment within the network.

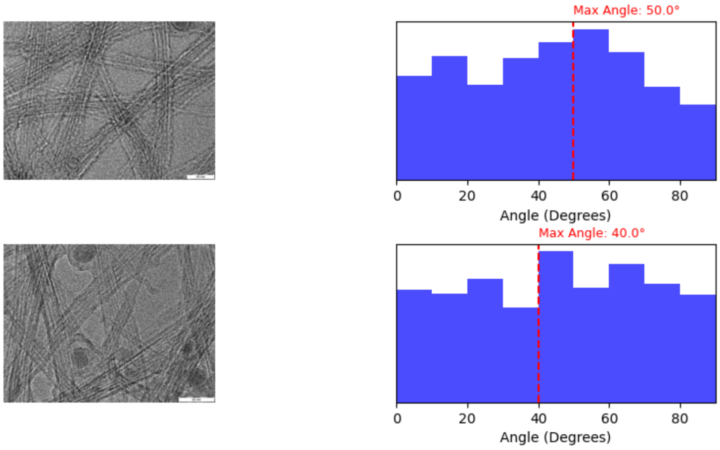

Fig. 10 Continue. Additional TEM images reveal the SWCNT network's structure. The angle distribution chart provides a statistical analysis of the SWCNTs' alignment within the network.

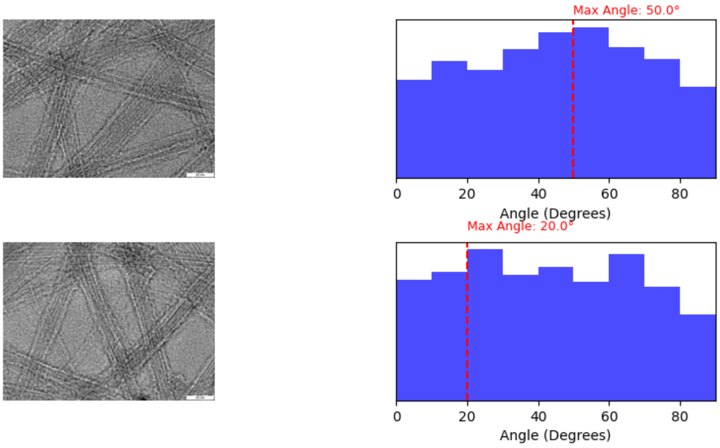

Fig. 11 Continue. Additional TEM images reveal the SWCNT network's structure. The angle distribution chart provides a statistical analysis of the SWCNTs' alignment within the network.

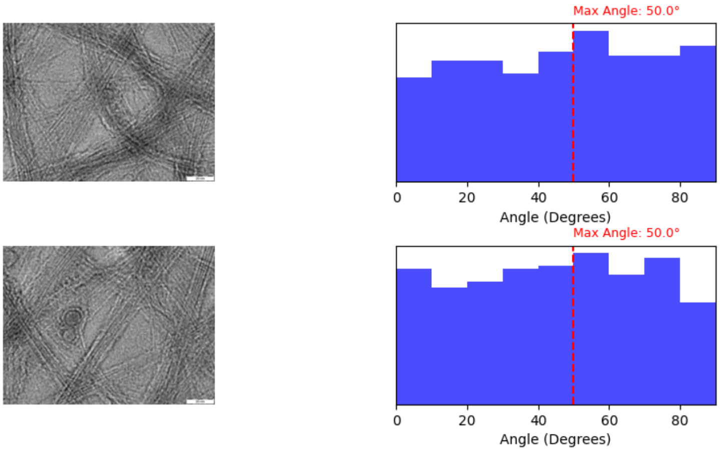

Fig. 12 Continue. Additional TEM images reveal the SWCNT network's structure. The angle distribution chart provides a statistical analysis of the SWCNTs' alignment within the network.

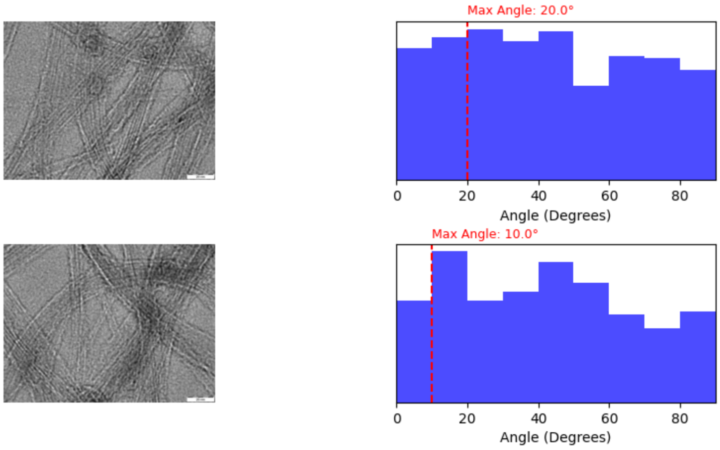

Fig. 13 Continue. Additional TEM images reveal the SWCNT network's structure. The angle distribution chart provides a statistical analysis of the SWCNTs' alignment within the network.

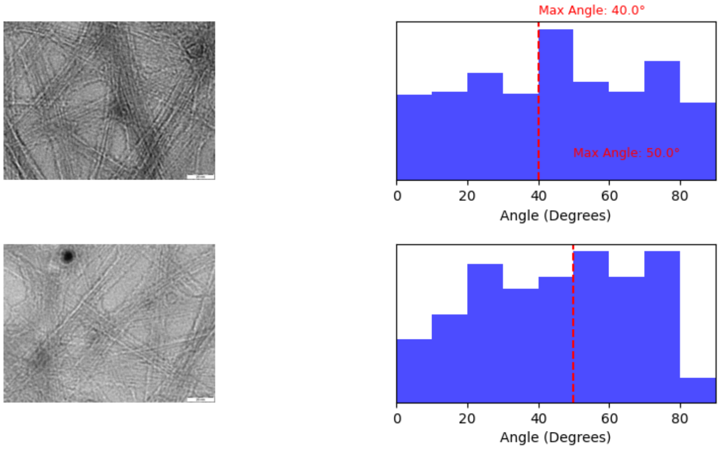

Fig. 14 Continue. Additional TEM images reveal the SWCNT network's structure. The angle distribution chart provides a statistical analysis of the SWCNTs' alignment within the network.

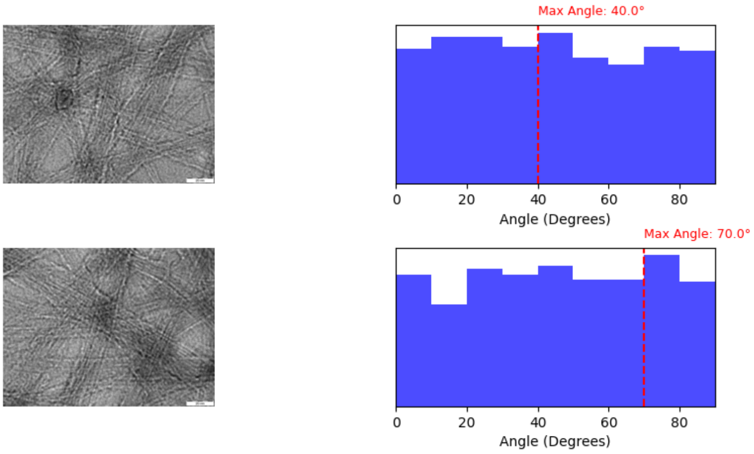

Fig. 15 Continue. Additional TEM images reveal the SWCNT network's structure. The angle distribution chart provides a statistical analysis of the SWCNTs' alignment within the network.

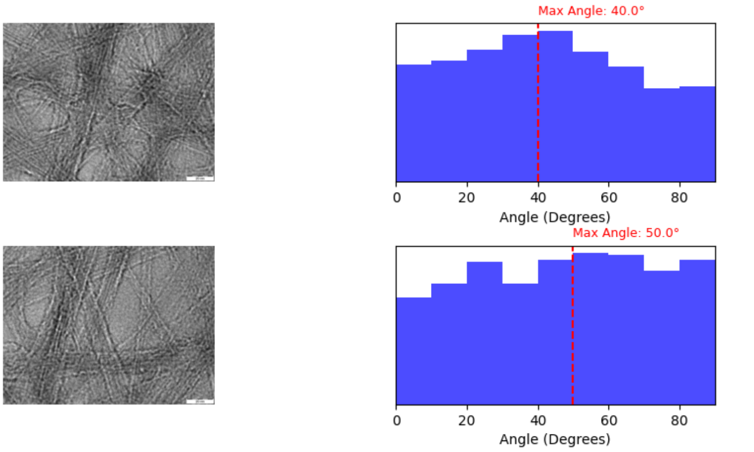

Fig. 16 Continue. Additional TEM images reveal the SWCNT network's structure. The angle distribution chart provides a statistical analysis of the SWCNTs' alignment within the network.

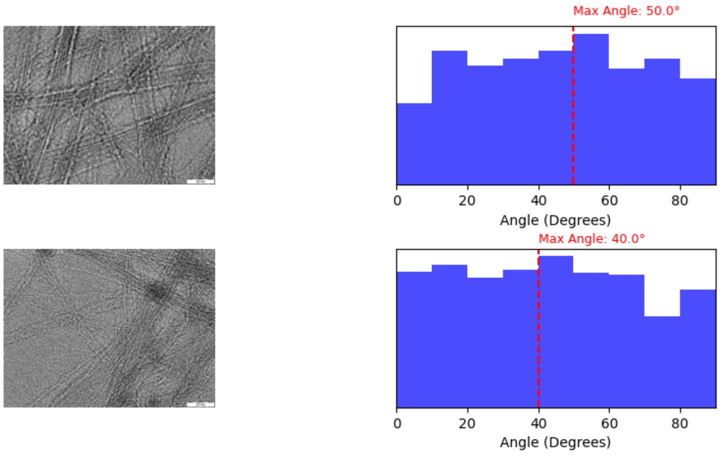

Fig. 17 Continue. Additional TEM images reveal the SWCNT network's structure. The angle distribution chart provides a statistical analysis of the SWCNTs' alignment within the network.

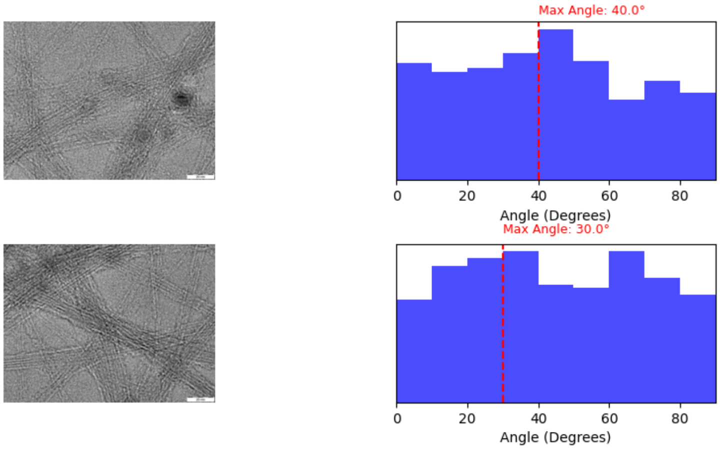

Fig. 18 Continue. Additional TEM images reveal the SWCNT network's structure. The angle distribution chart provides a statistical analysis of the SWCNTs' alignment within the network.

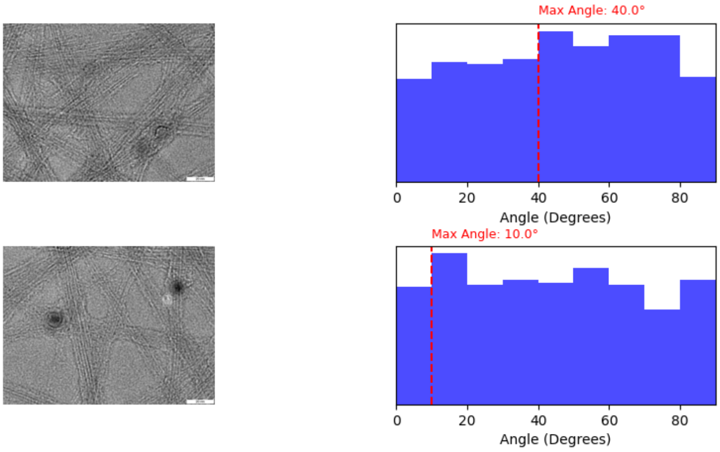

Fig. 19 Continue. Additional TEM images reveal the SWCNT network's structure. The angle distribution chart provides a statistical analysis of the SWCNTs' alignment within the network.

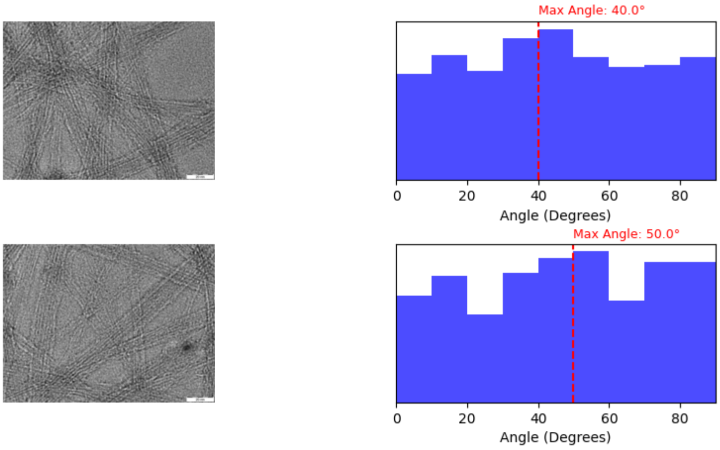

Fig. 20 Continue. Additional TEM images reveal the SWCNT network's structure. The angle distribution chart provides a statistical analysis of the SWCNTs' alignment within the network.

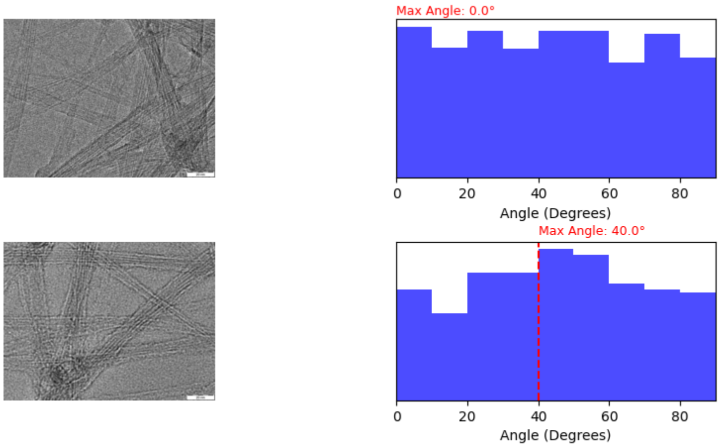

Fig. 21 Continue. Additional TEM images reveal the SWCNT network's structure. The angle distribution chart provides a statistical analysis of the SWCNTs' alignment within the network.

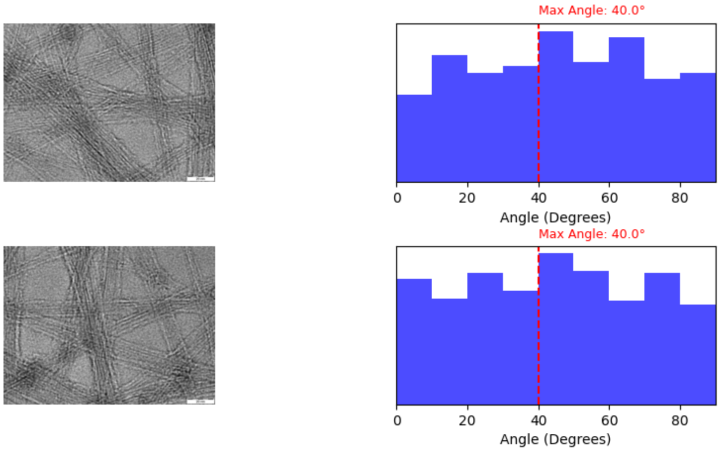

Fig. 22 Continue. Additional TEM images reveal the SWCNT network's structure. The angle distribution chart provides a statistical analysis of the SWCNTs' alignment within the network.

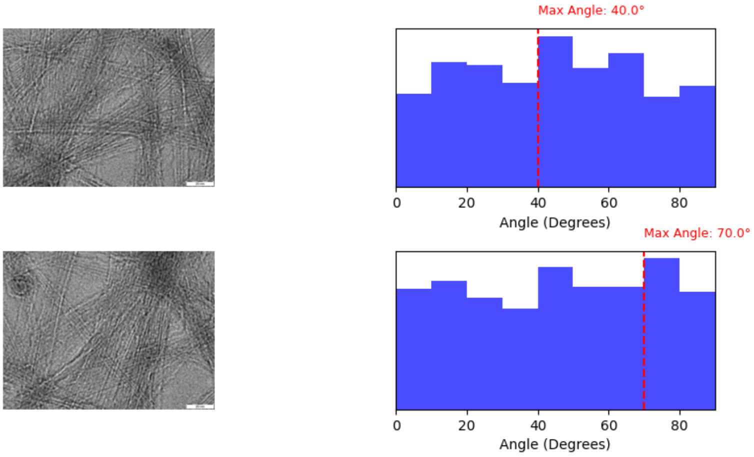

Fig. 23 Continue. Additional TEM images reveal the SWCNT network's structure. The angle distribution chart provides a statistical analysis of the SWCNTs' alignment within the network.

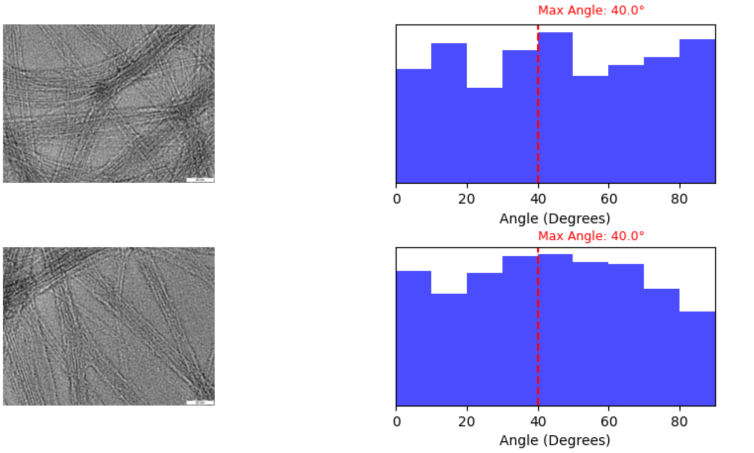

Fig. 24 Continue. Additional TEM images reveal the SWCNT network's structure. The angle distribution chart provides a statistical analysis of the SWCNTs' alignment within the network.

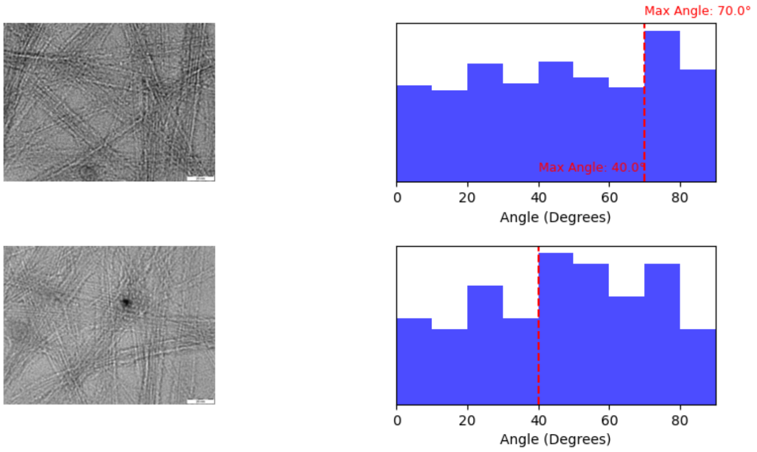

Fig. 25 Continue. Additional TEM images reveal the SWCNT network's structure. The angle distribution chart provides a statistical analysis of the SWCNTs' alignment within the network.

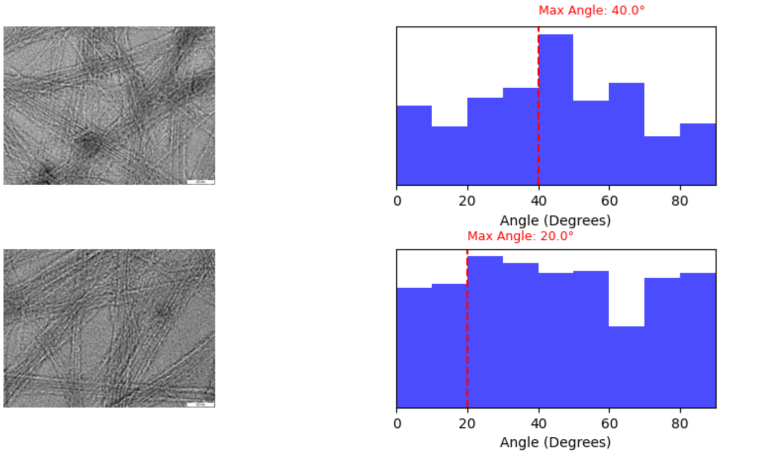

Fig. 26 Continue. Additional TEM images reveal the SWCNT network's structure. The angle distribution chart provides a statistical analysis of the SWCNTs' alignment within the network.

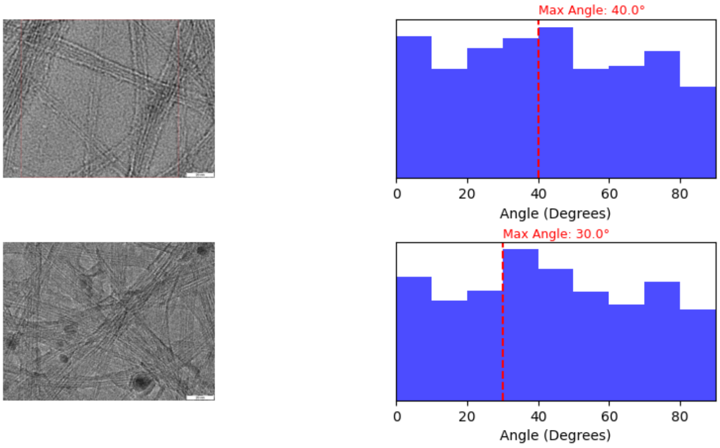

Fig. 27 Continue. Additional TEM images reveal the SWCNT network's structure. The angle distribution chart provides a statistical analysis of the SWCNTs' alignment within the network.

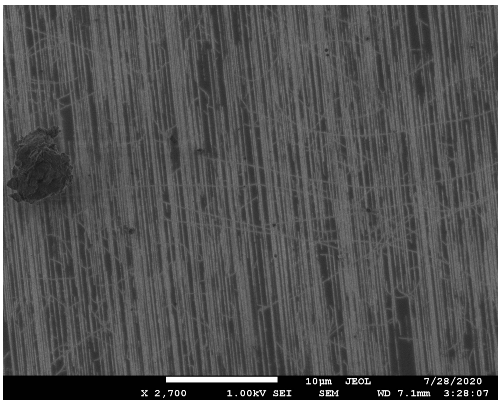

Fig. 28 Additional SEM images of aligned SWCNT network's structure.

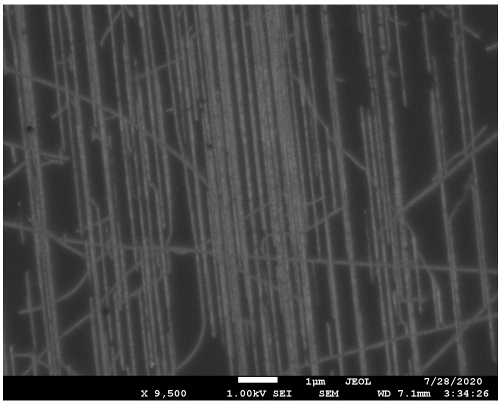

Fig. 29 Additional SEM images of aligned SWCNT network's structure.

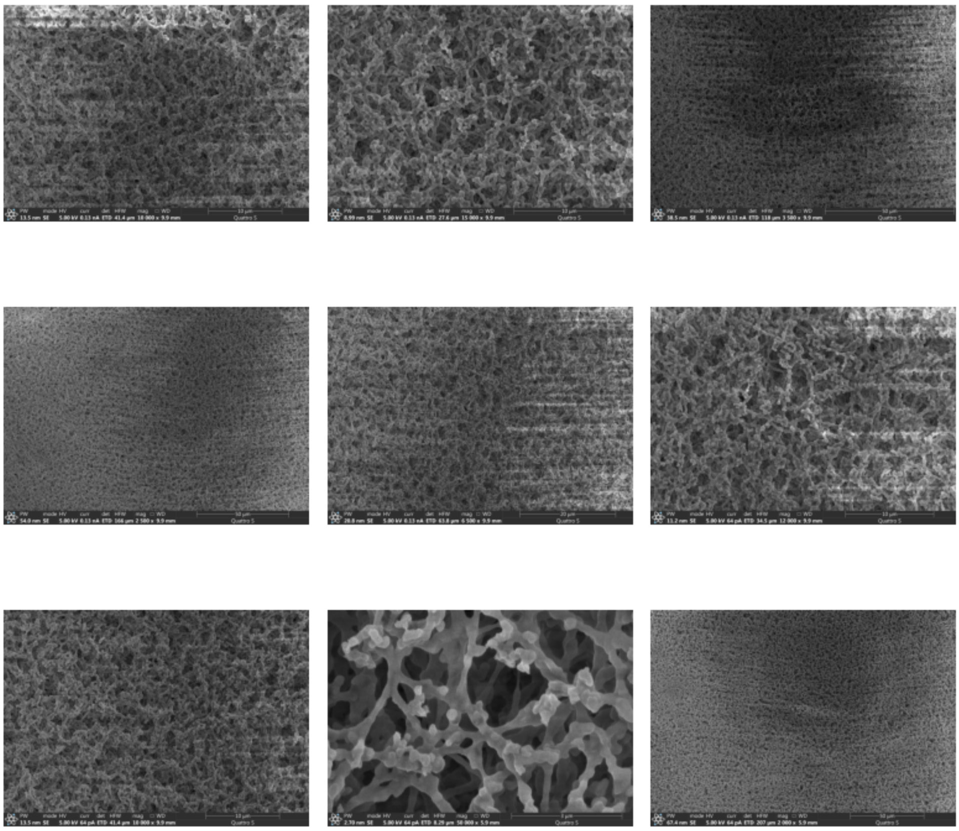

Fig. 30 SEM images of nitrocellulose membrane used in CNT synthesis.

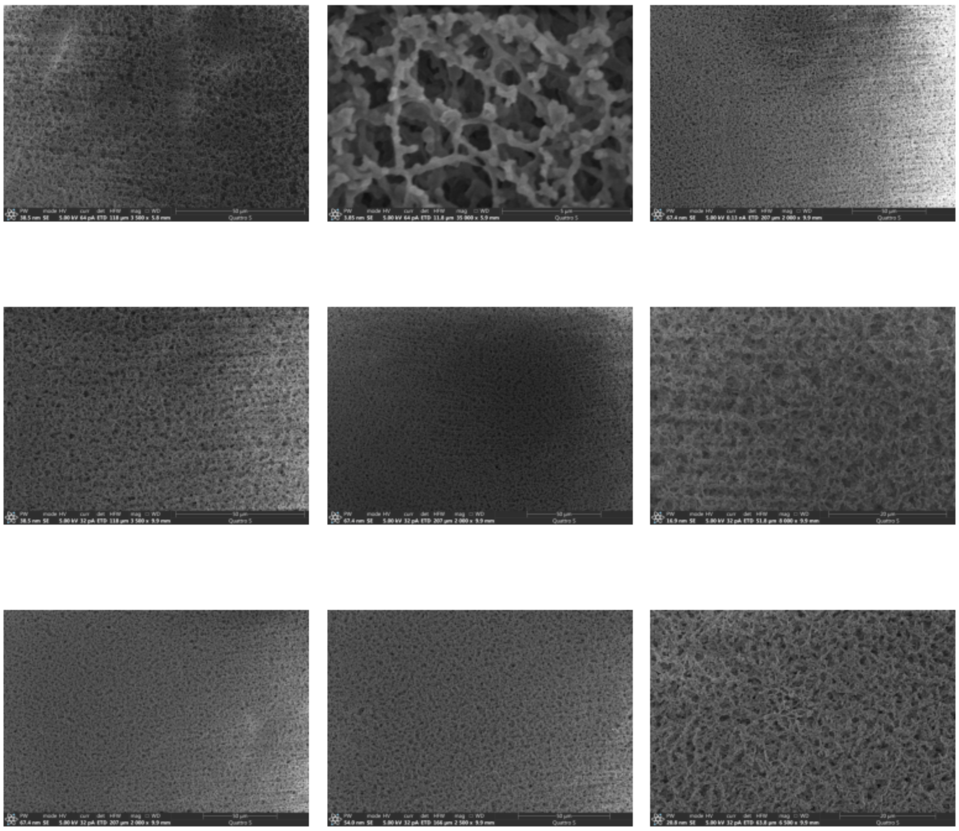

Fig. 31 Continue. SEM images of nitrocellulose membrane used in CNT synthesis.

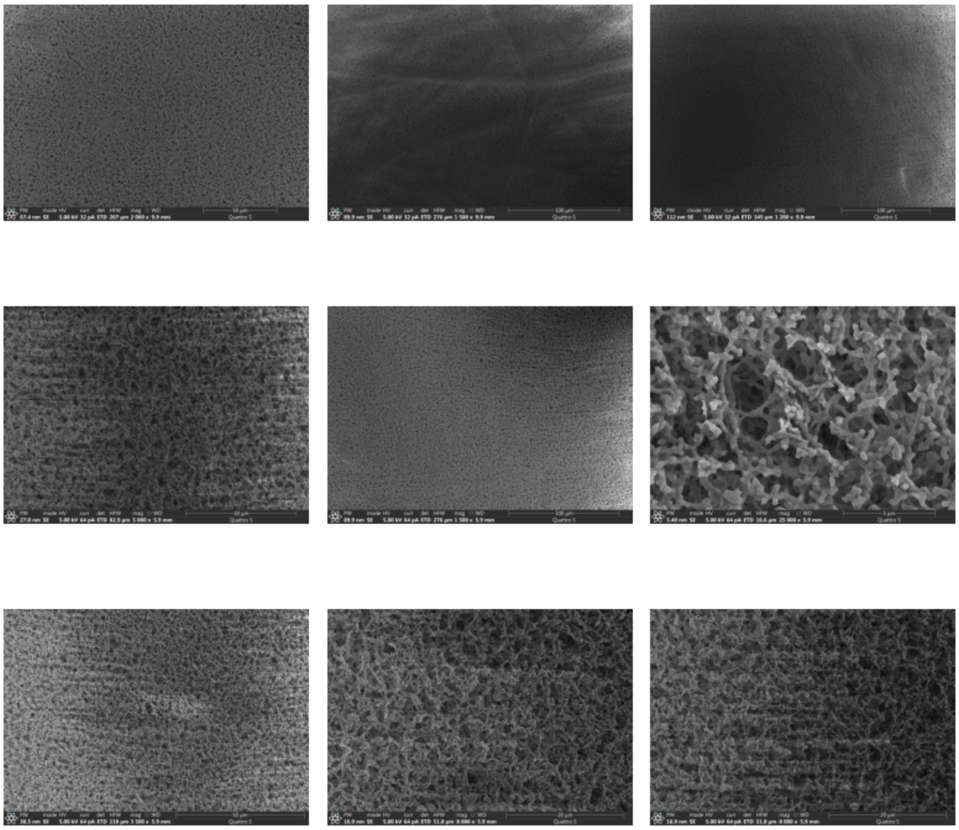

Fig. 32 Continue. SEM images of nitrocellulose membrane used in CNT synthesis.

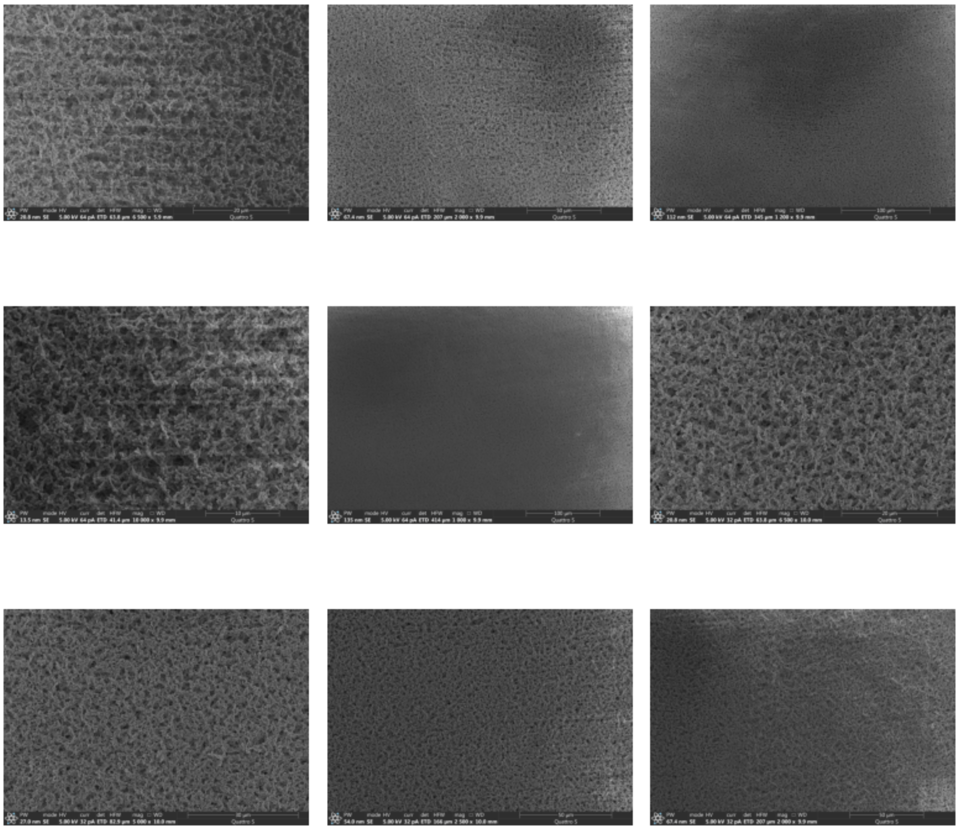

Fig. 33 Continue. SEM images of nitrocellulose membrane used in CNT synthesis.

References

[1] Khabushev, E. M. *et al.* Machine learning for tailoring optoelectronic properties of single-walled carbon nanotube films. *The Journal of Physical Chemistry Letters* **10**, 6962–6966 (2019). URL https://doi.org/10.1021/acs.jpclett.9b02777.

[2] Liu, W., Zhang, S., Qian, L., Lin, D. & Zhang, J. Growth of high-density horizontal swnt arrays using multi-cycle in-situ loading catalysts. *Carbon* **157**, 164–168 (2020). URL http://dx.doi.org/10.1016/j.carbon.2019.10.002.
